# Lifestyle and Clinical Predictors of Glial Cell Line-Derived Neurotrophic Factor Expression in Lumbosacral Stenosis-Related Ligamentum Flavum Degeneration

**DOI:** 10.3390/biomedicines13071530

**Published:** 2025-06-23

**Authors:** Dawid Sobański, Małgorzata Sobańska, Rafał Staszkiewicz, Damian Strojny, Werner Dammermann, Paweł Gogol, Weronika Wieczorek-Olcha, Artur Chwalba, Beniamin Oskar Grabarek

**Affiliations:** 1 Department of Neurosurgery, Szpital sw. Rafala in Cracow, 30-693 Cracow, Poland; sobanskamalgorzata05@gmail.com; 2 Collegium Medicum, WSB University, 41-300 Dabrowa Gornicza, Poland; rafalstaszkiewicz830@gmail.com (R.S.); drstrojny.ds@gmail.com (D.S.); drpawelgogol@gmail.com (P.G.); wercia092@gmail.com (W.W.-O.); bgrabarek7@gmail.com (B.O.G.); 3 Department of Neurosurgery, 5th Military Clinical Hospital with the SP ZOZ Polyclinic in Krakow, 30-901 Krakow, Poland; 4 Department of Neurosurgery, Faculty of Medicine in Zabrze, Academy of Silesia, 40-555 Katowice, Poland; 5 Institute of Health Care, National Academy of Applied Sciences in Przemyśl, 37-700 Przemyśl, Poland; 6 New Medical Techniques Specialist Hospital of St. Family in Rudna Mała, 36-060 Rzeszów, Poland; 7 Center of Internal Medicine II, University Hospital Brandenburg, 03048 Brandenburg, Germany; werner.dammermann@mhb-fontane.de; 8 Faculty of Health Sciences Brandenburg, Brandenburg Medical School Theodor Fontane, 16816 Neuruppin, Germany; 9 Department of Anesthesiology and Intensive Care, Our Lady of Perpetual Help Hospital in Wołomin, 05-200 Wołomin, Poland; 10 Department of Trauma and Orthopedic Surgery, Our Lady of Perpetual Help Hospital in Wołomin, 05-200 Wołomin, Poland; 11 Pain Treatment Clinic, Our Lady of Perpetual Help Hospital in Wołomin, 05-200 Wołomin, Poland; 12 General Rehabilitation Sub-Department, Edmund Wojtyla Małopolski Hospital for Lung Diseases and Rehabilitation, 32-310 Jaroszowiec, Poland; artur.adam.chwalba@gmail.com; 13 Department of Pharmacology, Faculty of Medical Sciences in Zabrze, Medical University of Silesia in Katowice, 41-808 Zabrze, Poland; 14 Faculty of Medicine and Health Sciences, Andrzej Frycz Modrzewski University, 30-705 Kraków, Poland

**Keywords:** glial cell line-derived neurotrophic factor (GDNF), degenerative stenosis, habits

## Abstract

**Background/Objectives**: Degenerative spinal conditions, such as degenerative stenosis, have been linked to metabolic and lifestyle factors, including obesity, smoking, and diabetes. Glial cell line-derived neurotrophic factor (GDNF) plays a crucial role in neuroprotection, but its relationship with these risk factors remains unclear. **Methods**: This study aims to evaluate the relationship between body mass index (BMI), smoking, diabetes, and GDNF levels in patients with degenerative spine conditions. We measured the GDNF levels in patients with degenerative stenosis and assessed the impact of BMI, smoking status, and the presence of diabetes. Comparisons were made using appropriate statistical analyses to determine the significance of these factors on GDNF levels. **Results**: A significant inverse relationship was observed between the BMI and GDNF levels (*p* < 0.01). Patients with a higher BMI exhibited lower GDNF concentrations. Additionally, patients who smoked or had diabetes showed significantly lower GDNF levels compared to non-smokers and those without diabetes (*p* = 0.03 and *p* = 0.02, respectively). These findings suggest that both metabolic and lifestyle factors are associated with decreased GDNF, which may accelerate neurodegenerative processes in the spine. **Conclusions**: Our study demonstrates that increased BMI, smoking, and diabetes are linked to reduced GDNF levels, potentially contributing to the progression of degenerative spine conditions such as stenosis. These findings highlight the need for targeted clinical interventions to manage these risk factors, aiming to preserve GDNF levels and slow the degenerative processes in the spine. Future research should explore therapeutic approaches to modulate GDNF in affected populations.

## 1. Introduction

Lumbosacral (L/S) spinal stenosis is characterized by a pathological narrowing of the spinal canal that results in compression of neural and vascular elements within the lumbar spine [1,2]. From a clinical standpoint, patients frequently report radiating pain extending to the buttocks and lower extremities, often accompanied by discomfort in the lower back [3,4]. A distinguishing clinical feature is neurogenic claudication—pain that intensifies with walking or prolonged standing and subsides when the patient is seated, reclined, or bends forward [4,5,6].

Anatomically, degenerative changes leading to spinal canal narrowing most frequently localize at the L4/L5 levels, likely due to its pivotal biomechanical role and high mobility [7].

Patients diagnosed with central or lateral recess stenosis may experience pain even at rest, during nighttime, or with actions such as coughing or sneezing. When neurological function remains preserved, conservative approaches, including physiotherapy and analgesics, are often effective. Surgical decompression is typically reserved for individuals with progressive pain, functional decline, or neurological deficits unresponsive to conservative management [8,9].

Surgical decompression, typically via laminectomy, aims to relieve pressure by removing hypertrophied ligament and enlarging the spinal canal [8,9]. Crucially, the pathophysiological mechanisms underlying lumbosacral spinal stenosis extend beyond mere mechanical compression; they are significantly modulated by complex molecular and inflammatory processes [10]. Emerging evidence highlights the involvement of various bioactive mediators in the development and maintenance of neuropathic pain in spinal degenerative disorders. Among these, brain-derived neurotrophic factor (BDNF) and glial cell line-derived neurotrophic factor (GDNF) are known to influence neuronal plasticity and survival, while substance P contributes to nociceptive signal transmission. Additionally, upregulation of pro-inflammatory markers such as cyclooxygenase-2 (COX-2), prostaglandin E2 (PGE2), and interleukin-6 (IL-6) further amplifies pain signaling pathways, suggesting a multifactorial molecular environment that exacerbates clinical symptoms [11,12]. Neurotrophins, in particular, have been widely explored for their roles in nociception, inflammatory signaling, and nerve regeneration [13].

However, to the best of our knowledge, the expression dynamics of GDNF within the human ligamentum flavum have not yet been systematically investigated, leaving a significant gap in understanding its potential role in the pathophysiology of lumbosacral spinal stenosis.

GDNF was first identified in 1993 when it was isolated from the supernatant of rat glial cells and found to have trophic effects on dopaminergic neurons in the midbrain. The gene responsible for encoding the GDNF protein is located on chromosome 5p13.2. Initially, GDNF was primarily recognized for its role in supporting the survival and function of dopaminergic neurons, which led to its application in gene therapy for Parkinson’s disease [14]. However, subsequent research has revealed its broader impact, affecting various neuron populations within the brain and the peripheral nervous system. GDNF also plays a crucial role in developmental processes such as kidney formation and spermatogenesis [15]. Its support of sympathetic, parasympathetic, sensory, and visceral neurons underscores its importance in maintaining the health and function of peripheral neural circuits [16].

Like BDNF [17,18], GDNF is also implicated in the modulation of pain signaling [19]. Despite this, there has been limited research on the specific role of GDNF in spinal cord degeneration and the discogenic pain associated with degenerative spine diseases, particularly in conditions such as lumbosacral stenosis [19]. However, findings from studies by Jung et al. demonstrated a marked increase in GDNF expression in both the dorsal horns of the spinal cord and the thalamus following spinal cord injury, suggesting its involvement in the body’s response to neural damage [20].

GDNF, a neurotrophic factor structurally related to the TGF-β superfamily, is predominantly expressed in both the central and peripheral nervous systems and plays a critical role in neuronal survival, differentiation, and regeneration [21]. Although it shares limited sequence similarity with TGFβ2, its biological functions are distinct. GDNF is secreted by various cell types—including Schwann cells, oligodendrocytes, astrocytes, motor neurons, and skeletal muscle—particularly during neural development [21]. Synthesized as an inactive precursor, it undergoes post-translational modifications to form an active homodimer [22,23,24].

Unlike classical TGFβ ligands, GDNF signals through a receptor complex involving the GPI-anchored GFRα1 or GFRα2 co-receptors and the RET tyrosine kinase receptor, which becomes activated via ligand-induced dimerization and autophosphorylation [22,25].

This study offers a novel exploration of the relationship between GDNF expression and lumbosacral spinal stenosis, an area that has not been extensively studied. By examining the changes in GDNF levels in patients suffering from lumbosacral stenosis, this research aims to provide new insights into the molecular mechanisms underlying this condition. This study highlights GDNF’s potential role in both the pain modulation and neuroregenerative processes associated with this degenerative disease, potentially opening new avenues for targeted therapies aimed at alleviating symptoms and promoting neural repair.

Therefore, this study aimed to assess GDNF concentration variability in the lumbosacral ligamentum flavum in relation to the degree of degeneration, pain severity, lifestyle factors, habits, and comorbid conditions.

## 2. Materials and Methods

This study was built upon the work conducted in our previous papers [26,27,28,29].

### 2.1. Characteristics of Study Group Participants

Ninety-six patients diagnosed with lumbosacral spinal stenosis were included in the study (46 women, 50 men; mean age: 68.3 ± 2.4 years). All were qualified for neurosurgical decompression via extended fenestration and foraminotomy. MRI confirmed the diagnosis and enabled an assessment of ligamentum flavum hypertrophy. The inclusion and exclusion criteria are summarized in Table 1.

#### Assessment of Clinical and Lifestyle Variables

Key clinical variables—including smoking status, glycemic control, alcohol consumption, and body mass index (BMI)—were assessed systematically at the time of participant enrollment. All data were obtained during structured preoperative interviews conducted by a qualified physician and cross-verified with the patients’ electronic medical records.

Smoking status was categorized as either “smoker” (current use of tobacco products at the time of admission) or “non-smoker.” Patients were asked explicitly about daily smoking habits and use of nicotine-containing products. Historical or former smoking was not considered in the “smoker” classification unless active use was reported within the past six months.

Glycemic control was determined by reviewing the diagnostic history, fasting plasma glucose levels, and glycated hemoglobin (HbA1c) results within the prior 3 months, if available. Patients were classified as diabetic if they had a confirmed diagnosis of diabetes mellitus (type 1 or 2) or met the World Health Organization (WHO) diagnostic criteria: HbA1c ≥ 6.5%, fasting glucose ≥ 126 mg/dL, or documented use of antidiabetic medication. A non-diabetic status was assigned to patients with normal fasting glucose and HbA1c values and no history of diabetes [30,31].

Alcohol consumption was assessed based on patient-reported intake, recorded as “yes” or “no.” Current alcohol use was defined as consumption of alcohol-containing beverages on at least a weekly basis within the past 3 months.

BMI was calculated as weight in kilograms divided by the square of height in meters (kg/m^2^) and categorized into normal weight (18.5–24.9), overweight (25.0–29.9), or obese (≥30.0) according to the WHO’s classifications [32].

### 2.2. Pain Assessment in Study Group

Pain intensity was documented using a 10-point Visual Analog Scale (VAS). None of the participants rated pain below 4. The most commonly reported levels were between 4 and 10. Specifically, pain ratings were as follows: 19 patients scored 4, 22 scored 5, 23 scored 6, 9 scored 7, 8 scored 8 or 9, and 7 reported a level of 10.

The VAS was selected as the primary pain assessment tool due to its simplicity, strong validation, and high sensitivity in capturing subtle differences in pain intensity. In comparison to alternative tools such as the Numeric Rating Scale (NRS) or Verbal Rating Scale (VRS), the VAS provides a continuous, non-categorical metric that minimizes the ceiling effects and clustering of responses, which is particularly advantageous for statistical analysis in clinical research. This decision is supported by existing literature. Hjermstad et al. [33] demonstrated that the VAS is a reliable and discriminative method for adult pain assessment in clinical studies, while Jensen et al. [34] showed that the VAS outperforms other methods in terms of sensitivity and responsiveness. Given our study’s aim to correlate patient-reported pain severity with molecular markers, the use of the VAS was deemed most appropriate for ensuring methodological robustness.

### 2.3. Description of Neurosurgical Procedure

All patients underwent decompressive spinal surgery consisting of extended fenestration and foraminotomy under general anesthesia. After localizing the affected level, the ligamentum flavum was resected to relieve neural compression. Microsurgical instruments were used throughout. In the absence of complications, patients were discharged on the third postoperative day and returned for follow-up after four weeks.

### 2.4. Characteristics of Control Group Participants

The control group consisted of 85 individuals (39 females, 46 males; mean age: 49.2 ± 2.7 years). Ligamentum flavum samples were collected postmortem from forensic autopsies or organ donors, following strict inclusion and exclusion criteria (Table 2). Tissue integrity was verified through hematoxylin and eosin (H&E) staining, and the final sample qualification was independently confirmed by two neurosurgeons (D.S. and R.S.).

### 2.5. Securing Collected Material for Molecular Testing

Following collection, ligamentum flavum specimens from both experimental and control cohorts were gently rinsed with a sterile buffer to remove residual blood or debris. Each sample was then transferred into sterile Eppendorf tubes prefilled with RNAlater stabilization solution (Invitrogen Life Technologies, Carlsbad, CA, USA) to preserve RNA integrity. The samples were subsequently frozen and stored at −80 °C until further processing for downstream molecular assays.

### 2.6. RNA Extraction and Assessment

The samples were homogenized using a T18 Digital Ultra-Turrax (IKA Polska Sp. z o.o., Warsaw, Poland) [35,36,37]. Total RNA was isolated using TRIzol reagent (Invitrogen Life Technologies, Carlsbad, CA, USA) and a modified phenol–chloroform protocol, followed by DNase I treatment and purification on RNeasy Mini columns (Qiagen, Valencia, CA, USA). RNA concentrations and purity were measured spectrophotometrically (NanoDrop^®^ instrument; Thermo Fisher Scientific, Waltham, MA, USA), and integrity was confirmed by gel electrophoresis, with clear 28S and 18S bands and A260/A280 ratios within the expected range.

### 2.7. Reverse Transcription Quantitative Polymerase Chain Reaction (RT-qPCR)

Quantification of gene expression was carried out using a one-step RT-qPCR protocol with Sensi-Fast Probe reagents (Bioline, London, UK). Reactions (50 µL) were run under standard cycling conditions, including reverse transcription at 45 °C and 40 amplification cycles. Target gene primers were as follows: forward 5′-GGCAGTGCTTCCTAGAAGAGA-3′, reverse 5′-AAGACACAACCCCGGTTTTTG-3′. GAPDH served as the reference gene (forward 5′-GGTGAAGGTCGGAGTCAACGGA-3′; reverse 5′-GAGGGATCTCGCTCCTGGAAGA-3′). Relative mRNA expression was calculated using the 2^−∆∆Ct^ method.

### 2.8. Protein Quantification Using Enzyme-Linked Immunosorbent Assay (ELISA) and Western Blotting Test

To evaluate the GDNF protein levels, ligamentum flavum specimens from both patient and control groups were processed for an enzyme-linked immunosorbent assay (ELISA) and Western blotting.

Ligamentum flavum samples from both groups were analyzed for GDNF protein levels using an ELISA and Western blot. For the ELISA, tissues were minced, incubated overnight at 4 °C in guanidine-based buffer (with protease inhibitors), and centrifuged. Supernatants were collected and stored at −20 °C. The GDNF concentration was determined with a polyclonal antibody (bs-1024R, STI, Poznań; 1:500) using HeLa lysates as the positive controls and omitting the primary antibody in the negative controls. Triplicate measurements were averaged for statistical analysis. The experimental conditions followed previously described protocols [38,39,40].

In parallel, Western blotting was performed. The samples were lysed in RIPA buffer, homogenized (Ultra-Turrax T18; IKA Poland Ltd., Warsaw, Poland), incubated on ice, and centrifuged. The protein content was quantified with a BCA assay (Thermo Fisher Scientific, Waltham, MA, USA), and equal protein amounts (20 µg) were resolved by SDS-PAGE (POL-AURA, Dywity, Poland) and transferred to PVDF membranes (pore size 0.45 µm, Thermo Fisher, USA). GDNF (~24 kDa) and β-actin (~42 kDa; loading control) were detected using specific antibodies (bs-1024R, 1:300; ACTB, Santa Cruz, Santa Cruz Biotechnology, Dallas, TX, USA 1:500). HRP-conjugated secondary antibodies (BioRad, Milan, Italy; 1:3000) enabled chemiluminescent detection. Band intensity was measured using Kodak MI 4.5SE software (Kodak, Rochester, NY, USA). Details of the immunodetection and validation steps are consistent with prior reports [29,38,39].

### 2.9. Immunohistochemical (IHC) Analysis

Tissue sections (8 µm) were cut using a rotary microtome (Leica Microsystems, Germany) and processed using standard paraffin-based IHC protocols with commercial kits (Vector Laboratories; Abcam, Newark, CA, USA). DAB was used for visualization. Staining was evaluated via light microscopy with a Nikon Coolpix system. For each subject, 15 representative fields (200× magnification) from three slides were analyzed using ImageJ software (ImageJ bundled with Java 8) with the IHC Profiler plugin [40,41]. Quantification was based on the DAB signal intensity and stained area percentage. Representative H&E images were previously published [39].

### 2.10. Statistical Analysis

Data analysis was conducted using Statplus (AnalystSoft, Brandon, FL, USA). Statistical significance was defined as *p* < 0.05. Normality was assessed using the Shapiro–Wilk test. Depending on the distribution, group comparisons were performed using either a Student’s *t*-test or one-way ANOVA, followed by Scheffé’s post hoc test when appropriate. Correlations between age and GDNF expression were evaluated using Pearson’s coefficient to identify potential confounding effects.

## 3. Results

### 3.1. Expression Changes at the mRNA GDNF in Control and Test Samples

**Figure 1:**
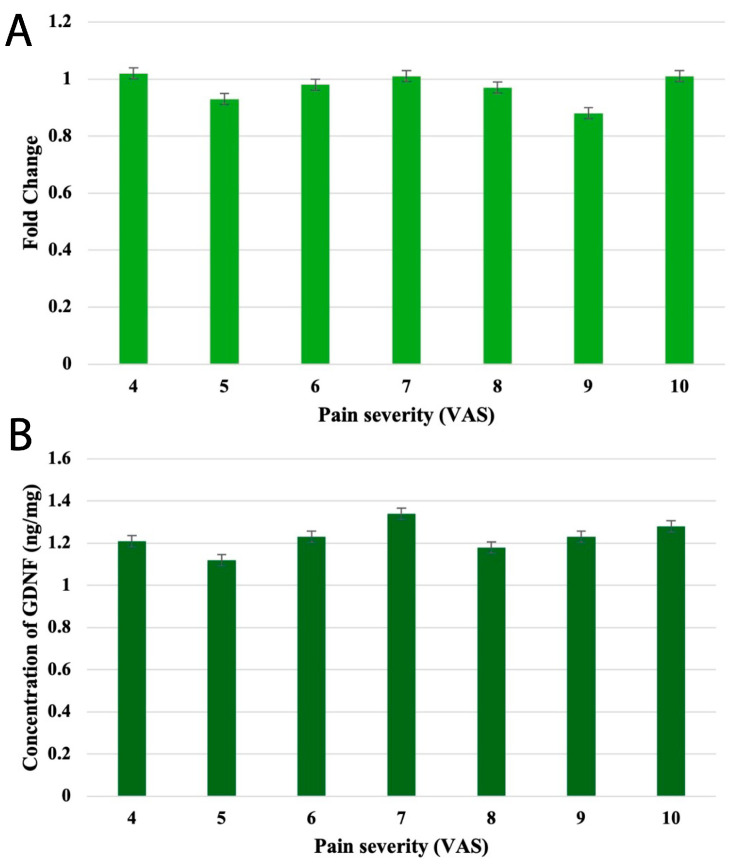
Expression profile of GDNF in degenerated yellow ligament tissue. (**A**) Relative *GDNF* mRNA expression levels assessed by RT-qPCR. (**B**) GDNF protein concentration measured by ELISA, stratified by VAS pain score categories.

RT-qPCR analysis demonstrated no statistically significant differences in GDNF mRNA expression between the study and control groups (Fold Change = 0.97 ± 0.10; *p* > 0.05). Subgroup analysis stratified by pain intensity also revealed no significant trends in the mRNA levels across the VAS categories (Figure 1A).

### 3.2. GDNF Protein Concentration by ELISA

Protein quantification via ELISA demonstrated a marked reduction in GDNF levels in the degenerative samples compared to the healthy controls (1.23 ± 0.45 ng/mg vs. 9.17 ± 1.11 ng/mg; *p* < 0.05). However, when stratified by pain intensity, GDNF concentrations did not significantly vary across the VAS categories 4 through 10 (Figure 1B; *p* > 0.05). The mean concentrations across pain levels showed minimal fluctuation, suggesting a pain-independent reduction in GDNF at the protein level. Additionally, Pearson’s correlation analysis indicated no meaningful association between the patient’s age and GDNF levels (r = 0.17; *p* = 0.29).

### 3.3. Validation of Protein Downregulation via Western Blot

**Figure 2:**
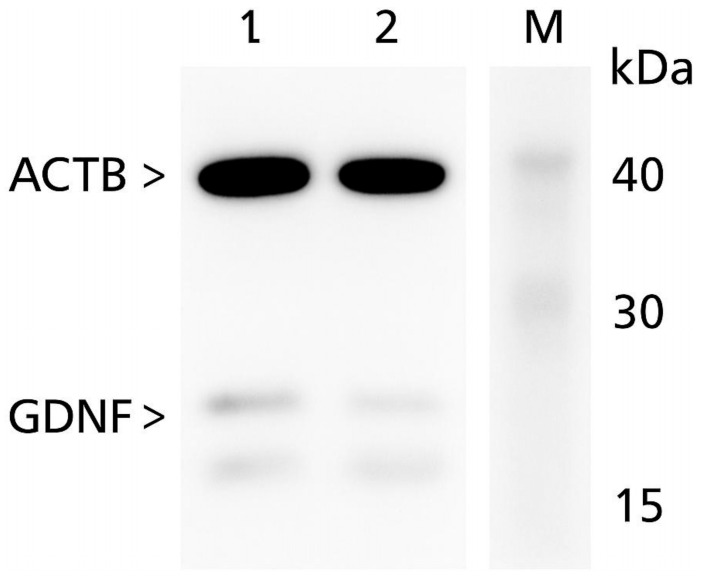
Representative Western blot analysis of GDNF protein expression in ligamentum flavum tissue. Lane 1—patient with ligamentum flavum degeneration (test); Lane 2—control subject without degenerative changes. GDNF, glial-derivied neurotrophic factor; ACTB, beta-actin; M, molecular weight marker.

Western blotting confirmed the ELISA findings. As shown in Figure 2, the control tissues exhibited substantially stronger GDNF bands (~24 kDa) relative to the study samples, with β-actin (~42 kDa) serving as the internal control. Densitometric analysis showed that GDNF band intensity normalized to ACTB was significantly diminished in the stenosis group (0.08 ± 0.03) versus the controls (8.12 ± 1.23; *p* < 0.05), corroborating the downregulation observed by the ELISA.

### 3.4. Influence of Lifestyle and Clinical Factors on GDNF Expression

We investigated whether the expression profiles of GDNF at both the mRNA and protein levels were influenced by gender, BMI, diabetes status, smoking, and alcohol consumption. The analysis revealed no significant differences in *GDNF* mRNA expression based on gender, diabetes, or alcohol consumption (Table 3; *p* > 0.05). However, significant differences in mRNA expression were observed for BMI and smoking (Table 3; *p* < 0.05). At the protein level, there were no significant differences based on gender or alcohol consumption (Table 3; *p* > 0.05), but significant differences were found in relation to BMI, diabetes, and smoking (Table 3; *p* < 0.05).

### 3.5. Regression Modeling of Predictors of GDNF Expression

The univariate regression analysis demonstrated no significant predictors of GDNF at the mRNA level. However, several clinical variables were significantly associated with the GDNF protein levels. Specifically, BMI (r = 0.61, *p* = 0.042), diabetes (r = 0.71, *p* = 0.001), smoking (r = 0.71, *p* = 0.012), and alcohol consumption (r = 0.91, *p* < 0.0001) were all correlated with protein expression. These findings were upheld in multivariate regression, where BMI (β = 0.176, *p* = 0.023), diabetes (β = 0.5456, *p* = 0.012), smoking (β = 0.312, *p* = 0.038), and alcohol use (β = 0.421, *p* < 0.0001) emerged as independent predictors of reduced GDNF protein content (Table 4).

### 3.6. Immunohistochemical Confirmation of GDNF Expression Patterns

**Figure 3:**
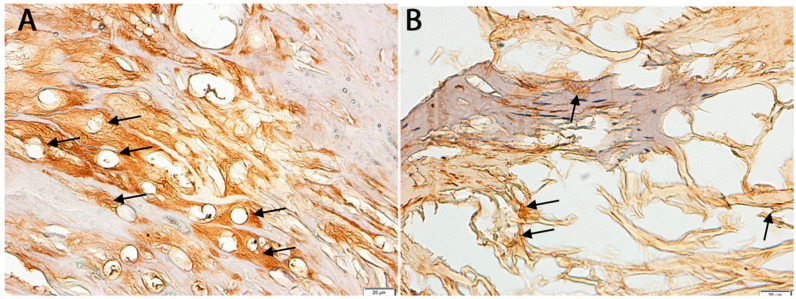
Examples of immunochemical expression of GDNF in the control (**A**) and study (**B**) samples. Arrows indicate areas of positive GDNF immunoreactivity in ligamentum flavum tissue (**A**) and control tissue (**B**), visualized using immunohistochemistry. Scale bar = 20 µm.

The HC analysis supported the molecular data, revealing visibly reduced GDNF staining intensity in the degenerated ligamentum flavum compared to the control tissues. Quantification showed an average optical density of 81.12% ± 3.45 in the stenosis samples relative to the controls (set at 100%), representing a significant reduction of 18.88% ± 2.98 (*p* < 0.05, Student’s *t*-test). The representative IHC images in Figure 3 highlight the decreased expression in patient samples (Figure 3B) versus the controls (Figure 3A).

## 4. Discussion

Degenerative lumbosacral spinal stenosis has become increasingly prevalent in modern societies, often resulting in significant functional impairment and contributing to high rates of disability and absenteeism from work [42,43]. As a result, there is growing interest in elucidating the biological mechanisms underlying symptom development in spinal degeneration, particularly those involved in pain generation and progression [44,45,46].

In this study, we focused on the expression profile of GDNF within ligamentum flavum tissue from patients with spinal stenosis. Although the *GDNF* mRNA levels did not differ significantly from those observed in the non-degenerative controls, a marked reduction in GDNF protein concentration was identified in affected tissue (*p* < 0.05). This apparent disconnect suggests that GDNF downregulation may not originate from transcriptional silencing but rather from post-transcriptional regulatory mechanisms.

One plausible explanation involves microRNA (miRNA)-mediated translational repression. Prior research by Shen et al. indicates that miR-204 can bind directly to the 3′ UTR of *GDNF* mRNA, hindering its translation without decreasing transcript abundance [47].

This mechanism could explain the discordance observed in our study, where GDNF *mRNA* expression remained relatively unchanged despite a significant drop in protein levels. Moreover, miR-204 expression has been shown to be modulated by oxidative stress and pro-inflammatory cytokines—both of which are elevated in degenerative spinal conditions—further supporting its pathological relevance in this context [48]. Beyond miR-204, other regulatory RNAs and RNA-binding proteins (RBPs) may also influence GDNF mRNA stability, localization, and translational efficiency. These layers of regulation underscore the complexity of neurotrophin control in degenerative diseases and highlight the importance of expanding future research to include miRNA profiling and proteomic-mRNA correlation analyses. Such approaches could clarify how translational repression contributes to GDNF depletion in spinal stenosis and potentially reveal novel targets for therapeutic intervention.

Despite the reduction in GDNF protein, no significant association was found between GDNF levels and pain severity, as measured by the visual analog scale (VAS). This should be interpreted cautiously, given the inherent limitations of subjective pain assessment tools [49,50].

To date, few studies have directly examined GDNF expression in ligamentum flavum or its potential role in degenerative spine disease [51,52,53].

GDNF, a member of the TGF-β superfamily, plays essential roles in neuronal maintenance, survival, and axonal extension [21]. Its role in peripheral and central nerve injury has been well documented. For instance, Jung et al. demonstrated GDNF upregulation in spinal cord tissues following injury, implicating it in neural protection and repair processes [20]. Additionally, GDNF contributes to peripheral nerve regeneration, partially by modulating inflammation through the suppression of cytokines, such as TNF-α and IL-1β, and by promoting ERK1/2 signaling to support neuronal viability [54,55]. Our data suggest that in ligamentum flavum tissue affected by degenerative changes, local GDNF protein availability is compromised, potentially limiting neurotrophic support and exacerbating tissue deterioration [56,57]. Interestingly, GDNF appears to behave differently depending on the tissue type and pathological context. While we observed decreased expression in ligament tissue, previous studies have reported elevated GDNF levels in degenerated intervertebral discs [38,58]. These opposing patterns may reflect tissue-specific regulation or divergent roles of GDNF in neuronal versus connective components of the spinal axis.

We also evaluated how patient-specific factors—particularly lifestyle and metabolic variables—affect GDNF expression. In agreement with prior literature, we found that smoking was significantly associated with lower GDNF protein levels [59,60,61]. Cigarette smoke may impair neurotrophic signaling through oxidative injury and chronic systemic inflammation. This aligns with clinical observations linking smoking to worse spinal degeneration and heightened pain sensitivity [62,63,64]. Obesity similarly correlated with reduced GDNF levels in ligamentum flavum. Chronic low-grade inflammation and biomechanical stress that is induced by excess body weight may disrupt tissue integrity and neurotrophic balance [65,66]. Evidence suggests that these two factors—smoking and obesity—interact to worsen surgical outcomes, prolong recovery, and increase neurological deficits in spinal stenosis [67]. Diabetes mellitus was another factor significantly linked to decreased GDNF protein expression in our cohort. Hyperglycemia and associated mitochondrial dysfunction may downregulate GDNF expression through enhanced oxidative stress [68,69]. Interestingly, experimental models suggest that the central overexpression of GDNF—particularly in dopaminergic circuits—can affect body weight and feeding behavior, linking it to energy homeostasis, as well [70,71]. This dual role in both neural and metabolic systems underscores the broader physiological relevance of GDNF.

Taken together, our findings reveal that GDNF expression in degenerative spinal tissue is subject to multifactorial regulation. Notably, body mass index, smoking status, and diabetic status all negatively impacted GDNF protein levels, suggesting that lifestyle modification may represent an indirect means of preserving neurotrophic function in spinal disease.

From a clinical standpoint, the diminished GDNF availability in ligamentum flavum could impair local neural repair mechanisms, contributing to disease progression. Strategies aimed at restoring or enhancing GDNF function may, therefore, hold promise as adjunctive treatments in patients with high-risk metabolic or lifestyle profiles.

One notable limitation of this study is the age difference between the study group (mean age: 68 years) and the control group (mean age: 49 years). This discrepancy reflects the limited availability of asymptomatic, non-degenerative ligamentum flavum tissues from elderly individuals undergoing surgery for non-degenerative indications. Given that age-related changes in gene expression, including GDNF, have been reported in the literature, age could act as a potential confounding factor.

To assess the impact of age on GDNF expression, we performed a Pearson’s correlation analysis across the entire cohort. The analysis revealed only a weak, statistically non-significant correlation between age and GDNF levels (r = 0.17, *p* = 0.29), suggesting that age is unlikely to be the primary driver of the observed differences. Nonetheless, we acknowledge this limitation, and future studies with age-matched control groups or age-adjusted models are warranted to further validate these findings.

Additionally, this study’s sample size, particularly for the control group, may affect the generalizability of the findings. Moreover, the reliance on subjective pain scales, like the VAS, to assess pain intensity introduces potential bias, as no objective pain measurement tools are currently available. Finally, lifestyle factors such as smoking and metabolic diseases, while evaluated, were self-reported, which may also introduce variability or inaccuracies in the data. Although post-transcriptional regulation by miR-204 was suggested as a potential mechanism for reduced GDNF protein levels, this study did not directly measure miRNA expression, which limits our mechanistic insight into miRNA-mediated repression. Future studies should consider larger cohorts, objective pain assessment methods, and improved tissue analysis techniques to address these limitations.

## 5. Conclusions

This study demonstrates a significant reduction in GDNF protein levels in the ligamentum flavum of patients with lumbosacral spinal stenosis, which appears to be influenced by key modifiable factors such as obesity, diabetes mellitus, and smoking. Notably, this downregulation was observed at the protein level without a corresponding decrease in *GDNF* mRNA expression, suggesting that post-transcriptional regulatory mechanisms play a critical role in modulating GDNF availability in degenerative spinal pathology.

The observed discrepancy between mRNA and protein levels underscores the potential involvement of miRNAs, RNA-binding proteins, and proteasomal degradation pathways in the regulation of GDNF expression. Additionally, chronic systemic inflammation associated with metabolic syndrome and smoking may influence signaling pathways such as NF-κB, PI3K/AKT, and mTOR, which are known to affect the translation efficiency and stability of neurotrophic factors.

Another potential layer of regulation involves oxidative stress-induced modification of translational machinery and the activation of stress granules, which could further impair the translation of *GDNF* mRNA under pathophysiological conditions. Furthermore, protein degradation pathways, including the ubiquitin-proteasome system, may contribute to accelerated GDNF turnover in degenerative tissues, especially in response to elevated levels of pro-inflammatory cytokines and reactive oxygen species.

These findings highlight the necessity of integrating transcriptomic, epigenetic, and proteomic analyses to fully elucidate the mechanisms underlying GDNF dysregulation in spinal degenerative diseases. Future research should prioritize the identification of specific miRNAs and post-transcriptional repressors of GDNF, the assessment of protein degradation dynamics, and the characterization of tissue-specific signaling networks that converge on neurotrophic factor regulation. Moreover, longitudinal studies are needed to explore how lifestyle interventions or pharmacological modulation of miRNA expression and proteostasis might restore GDNF function and alleviate clinical symptoms in affected individuals.

In conclusion, our study provides a molecular framework that connects lifestyle-related risk factors with impaired neurotrophic signaling in the ligamentum flavum. A deeper understanding of the post-transcriptional control of GDNF may offer new diagnostic biomarkers and therapeutic targets for the management of spinal stenosis and related degenerative disorders.

## Figures and Tables

**Table 1:** Inclusion and exclusion criteria for the study group [26,27,28,29].

Inclusion Criteria	Exclusion Criteria
Confirmation of degenerative stenosis of the lumbosacral spine in imaging studies	Exclusion of degenerative stenosis of the lumbosacral spine on imaging studies
Caucasian race	Race other than Caucasian
Age ≥18 and ≤80 years	Age <18 or >80 years
No serious contraindications to surgical or internal medicine treatment	Serious contraindications to surgical or internal medicine treatment
Not taking anticoagulants or discontinuing as recommended after consultation	Taking anticoagulants and inability to discontinue them safely
Ineffective conservative treatment for ≥6 months	Effective conservative treatment
Presence of neurological symptoms (e.g., radiculopathy, claudication)	History of previous surgical treatment at the lumbosacral spine level
No intake of vitamin and mineral preparations registered as medication in the last 6 months	Intake of vitamin and mineral preparations registered as medication in the last 6 months
No history of decompensated hormonal disorders	Presence of unbalanced hormonal disorders
No gastrointestinal disorders	Gastrointestinal disorders, including malabsorption syndromes
Not pregnant	Pregnant
Not lactating	Lactating
Not menstruating at the time of surgery (outside of bleeding phase or immediately after)	Menstruating during or immediately after bleeding phase

**Table 2:** Inclusion and exclusion criteria for the control group [26,27,28,29].

Inclusion Criteria	Exclusion Criteria
Provided informed, voluntary consent	Lack of informed, voluntary consent
Age ≥18 and <80 years	Age <18 or ≥80 years
No history or current diagnosis of neoplastic diseases	Current or past neoplastic diseases
No history of degenerative spine disease or spinal trauma, particularly in the L/S region	Presence or history of degenerative spinal disease and/or spinal trauma, especially in the L/S region
No use of vitamin or mineral supplements registered as medicinal products in past 6 months	Use of vitamin or mineral supplements registered as medicinal products within the past 6 months
No history of hormonal disorders	Presence of unbalanced hormonal disorders
No history of gastrointestinal disorders, including malabsorption	History of gastrointestinal disorders, including malabsorption
Not pregnant	Pregnant
Not lactating	Lactating
In a phase of the menstrual cycle outside of active bleeding or immediately post-bleeding	Experiencing menstrual bleeding or within the immediate post-bleeding phase

**Table 3:** The expression of GDNF at the mRNA and protein levels in ligamentum flavum samples obtained from the study group.

Comparison		mRNA	Student’s *t*-Test ^1^ or ANOVA ^2^ (Study Group)	Protein	Student’s *t*-Test ^1^ or ANOVA ^2^ (Control Group)
Gender	Female (*n* = 46)	0.95 ± 0.09	0.987 ^1^	1.27 ± 0.19	0.432 ^1^
	Male (*n* = 50)	0.99 ± 0.02		1.19 ± 0.21	
BMI (kg/m^2^)	Normal (*n* = 40)	1	0.048 ^2^	1	0.032 ^2^
	Overweight (*n* = 32)	0.99 ±0.10		1.06 ±0.33	
	Obesity (*n* = 24)	0.91± 0.05		1.01± 0.24	
Diabetes	No (*n* = 46)	0.92 ± 0.11	0.765 ^1^	1.17 ± 0.21	0.042 ^1^
	Yes (*n* = 40)	1.02 ± 0.12		1.29 ± 0.32	
Smoking	No (*n* = 34)	1.09 ± 0.21	0.0412 ^1^	1.41 ± 0.21	0.039 ^1^
	Yes (*n* = 62)	0.85 ± 0.11		1.04 ± 0.18	
Drinking alcohol	No (*n* = 11)	0.98 ± 0.04	0.253 ^1^	1.02 ± 0.12	0.045 ^1^
	Yes (*n* = 85)	0.96 ± 0.02		1.43 ± 0.12	

GDNF, glial-derived neurotrophic factor; BMI, body mass index; r, correlation coefficient. Variables found to be insignificant using linear regression were not included in the multiple regression model.

**Table 4:** Univariate and multivariate regression analyses of the variables that may be associated with GDNF levels determined in the degenerative yellow ligamentum flavum.

Characteristic	Expression Level	Linear Regression			Multiple Regression	
		r	R^2^	*p*-Value	Coefficient	*p*-Value
Gender	mRNA	0.18	0.03	0.432		
	Protein	0.21	0.04	0.312		
BMI (kg/m^2^)	mRNA	0.19	0.04	0.617		
	Protein	0.61	0.37	0.042	0.176	0.023
Diabetes	mRNA	0.23	0.05			
	Protein	0.71	0.50	0.001	0.5456	0.012
Smoking	mRNA	0.23	0.05	0.512		
	Protein	0.71	0.50	0.012	0.312	0.038
Drinking alcohol	mRNA	0.19	0.04	0.871		
	Protein	0.91	0.83	<0.0001	0.421	<0.0001

BDNF, brain-derived neurotrophic factor; BMI, body mass index; r, correlation coefficient. R^2^, coefficient of determination. Variables found to be insignificant using linear regression were not included in the multiple regression model.

## Data Availability

The data used to support the findings of this study are included in the article. The data cannot be shared due to third-party rights and commercial confidentiality.
